# Low‐intensity shockwave therapy for erectile dysfunction due to diabetes mellitus or coronary artery disease: An individual participant data meta‐analysis from a single center

**DOI:** 10.1111/andr.70069

**Published:** 2025-05-22

**Authors:** Nikolaos Pyrgidis, Dimitrios Kalyvianakis, Ioannis Mykoniatis, Dimitrios Hatzichristou

**Affiliations:** ^1^ Department of Urology University Hospital LMU Munich Munich Germany; ^2^ Institute for the Study of Urological Diseases University of Thessaloniki Thessaloniki Greece; ^3^ Department of Urology Aristotle University of Thessaloniki Thessaloniki Greece

**Keywords:** coronary artery disease, diabetes mellitus, erectile dysfunction, erectile function, low‐intensity shockwave therapy

## Abstract

Background

Low‐intensity shockwave therapy (LiST) is a first‐line treatment for vasculogenic erectile dysfunction (ED). However, its efficacy in challenging cases, such as ED solely due to type 2 diabetes mellitus (DM) or coronary artery disease (CAD), has not been adequately assessed.

**Materials and methods:**

This study presents an individual participant data meta‐analysis of five double‐blind randomized controlled trials involving 208 patients treated in a single academic center using a standardized LiST protocol (ARIES 2 generator, 5000 impulses/session).

**Results:**

For outcomes, including International Index of Erectile Function‐Erectile Function Domain scores, sexual encounter profile question 3 responses, resistance index, and minimal clinically important differences, LiST was equally effective in patients with ED due to DM or CAD compared to other causes. Subgroup analyses showed equivalent efficacy for 6 versus 12 LiST sessions. No adverse events were reported.

**Conclusions:**

These findings confirm LiST's safety and effectiveness across different causes of vasculogenic ED, supporting its broader application in clinical practice.

Low‐intensity shockwave therapy (LiST) is currently recommended as a first‐line treatment modality for carefully selected patients with vasculogenic erectile dysfunction (ED).^1^ The randomized controlled trials (RCTs) that formed the basis for this recommendation included patients with different causes of vasculogenic ED.^2^ Among these causes, type 2 diabetes mellitus (DM) and coronary artery disease (CAD) are considered the most challenging.^3^

DM is associated with endothelial dysfunction, leading to macrovascular and microvascular impairment of the erectile tissue.^4^ Accordingly, in patients with CAD, ED is not only caused by endothelial dysfunction but also by the medications that are commonly prescribed for its management.^5^ Studies on phosphodiesterase 5 inhibitors (PDE5i) indicate that PDE5i are equally safe and effective in patients with DM or CAD compared to other causes of vasculogenic ED.^6^ Nevertheless, studies on the interplay between these major causes of ED and LiST are lacking. To address this gap, we conducted the first individual participant data (IPD) meta‐analysis on the role of LiST for vasculogenic ED due to DM or CAD.

An IPD meta‐analysis is particularly valuable in this context as it allows for standardized outcome assessment across studies, adjustment for participant‐level covariates, and more precise subgroup analyses, which are essential when evaluating treatment efficacy in complex populations such as patients with vasculogenic ED due to DM or CAD. We included all prior clinical trial participants who were randomized to receive LiST in terms of a double‐blind RCT in our Academic Department of Urology. The prespecified inclusion criteria for all RCTs in our center were: (i) sexually active males in a stable, heterosexual relationship for at least 3 months; (ii) age between 40 and 70 years; (iii) presence of vasculogenic ED (based on medical history) with regular use of any PDE5i and; (iv) agreement to suspend any ED treatment for the duration of each RCT.

All patients underwent the same LiST protocol regarding selection criteria, LiST generator (ARIES 2 with the Smart Focus probe provided by Dornier MedTech GmbH, Wessling, Germany) and impulses (2000 to the corpus, 2000 to the crura, and 1000 to the penile hila). Subsequently, these patients were assessed with the International Index of Erectile Function—Erectile Function Domain (IIEF‐EF), and the proportion of “yes” responses to question 3 of the Sexual Encounter Profile diaries (SEPQ3) at 1, 3, and 6 months after completion of the treatment protocol. Moreover, penile hemodynamics with ultrasonography (resistance index) were performed at 3 months.

The outcomes of this study were to evaluate the effect of LiST in patients with ED due to DM or CAD assessed with the IIEF‐EF, SEPQ3 (at 1, 3, and 6 months after completion of the treatment protocol), and resistance index (at 3 months), as well as the number of patients attaining a minimal clinically important difference (MCID) of erectile function improvement based on the IIEF‐EF.^7^ For these outcomes, we calculated mean differences or odd rations with their 95% confidence intervals, which were then pooled across trials with a stratified‐by‐trial, two‐stage, fixed‐effects IPD meta‐analysis. A one‐staged approach was also used to evaluate the robustness of our results. Heterogeneity was assessed with the χ^2^. We also performed a subgroup analysis to estimate the effect of LiST based on the number of sessions (6 versus 12). All statistical analyses were performed with the R software (version 3.6.3).

We included five RCTs published by our research group.^8^, ^9^, ^10^, ^11^, ^12^ All studies were considered at low risk of bias based on the RoB‐2. Ultimately, 208 patients with a median age of 57 (Interquartile range: 51–62) years were enrolled. Of them, 50 (24%) had ED due to DM and 28 (13%) due to CAD. Their ED duration was 282 (Interquartile range: 36–408) months, and their grade of ED was mild in 94 (45%) cases, moderate in 70 (34%) cases, and severe in 44 (21%) cases. PDE5i intake in the form of daily 5 mg tadalafil was allowed in two studies^10^, ^12^ (Table 1). In the IPD meta‐analysis, no differences were observed between patients with ED due to DM versus no DM in terms of IIEF‐EF, MCID, SEPQ3, and resistance index. Similarly, for these outcomes LiST was equally effective in patients with ED due to CAD versus no CAD (Table 2).

**TABLE 1:** The characteristics of the included studies.

First author, year of publication	Population	Treatment protocol	Participants (n)	Follow‐up evaluation (months)
**Kalyvianakis 2018** ^8^	All grades of vasculogenic ED (IIEF‐EF: < 26)	Group A: 6 LiST sessions (once/week for 6 weeks) Group B: 12 LiST sessions (twice/week for 6 weeks)	Group A: 21 Group B: 21	1, 3, 6, and 12
**Kalyvianakis 2020** ^9^	All grades of vasculogenic ED (IIEF‐EF: < 26)	Group A: 12 LiST sessions (twice/week for 6 weeks) Group B: 12 LiST sessions (three times/week for 4 weeks)	Group A: 24 Group B: 24	1, 3 and 6
**Mykoniatis 2022** ^10^	Mild vasculogenic ED (IIEF‐EF: 17–25)	Group A: 6 LiST sessions (twice/week for 3 weeks) + 5 mg tadalafil (for 4 weeks) Group B: 6 LiST sessions (twice/week for 3 weeks) + placebo (for 4 weeks)	Group A: 25 Group B: 25	1, 3 and 6
**Kalyvianakis 2022** ^11^	Moderate vasculogenic ED (IIEF‐EF: 11–16)	Group A: 12 LiST sessions (twice/week for 6 weeks)	Group A: 34	1, 3
**Kalyvianakis 2024** ^12^	Severe vasculogenic ED (IIEF‐EF: 1–10)	Group A: 12 LiST sessions (three times/week for 4 weeks) + 5 mg tadalafil (for 4 weeks)	Group A: 34	1, 3

Abbreviations: ED, erectile dysfunction; IIEF‐EF, International Index of Erectile Function‐Erectile Function; LiST: low‐intensity shockwave therapy.

**TABLE 2:** Outcomes of low‐intensity shockwave therapy in patients with DM vs. no DM, as well as in patients with CAD vs. no CAD.

Group	Outcome	Number of patients (active vs. comparator	Effect estimate	95% Confidence interval, heterogeneity *I* ^2^
**DM vs. No DM**	**IIEF‐EF at 1 month**	50 vs. 158	MD: 0.18	−0.53 to 0.89, 23%
	**IIEF‐EF at 3 months**	50 vs. 156	MD: 0.03	−0.71 to 0.76, 0%
	**IIEF‐EF at 6 months**	34 vs. 102	MD: 0.54	−0.57 to 1.64, 0%
	**MCID at 1 month**	25/50 vs. 102/158	OR: 0.6	0.28 to 1.25, 0%
	**MCID at 3 months**	28/50 vs. 113/156	OR: 0.55	0.26 to 1.16, 0%
	**MCID at 6 months**	24/34 vs. 88/102	OR: 0.54	0.21 to 1.44, 0%
	**SEPQ3 at 1 month**	50 vs. 158	MD: 7	−1.3 to 15, 47%
	**SEPQ3 at 3 months**	50 vs. 156	MD: 1.7	−6.6 to 9.9, 18%
	**SEPQ3 at 6 months**	34 vs. 102	MD: 2.4	−8.9 to 14, 4%
	**RI at 3 months**	30 vs. 58	MD: −0.02	−0.08 to 0.03, 0%
**CAD versus No CAD**	**IIEF‐EF at 1 month**	28 vs. 180	MD: −0.46	−1.17 to 0.24, 0%
	**IIEF‐EF at 3 months**	28 vs. 178	MD: −0.05	−0.59 to 0.5, 63%
	**IIEF‐EF at 6 months**	13 vs. 123	MD: −0.53	−1.94 to 0.89, 0%
	**MCID at 1 month**	12/28 vs. 115/180	OR: 0.6	0.24 to 1.5, 0%
	**MCID at 3 months**	13/28 vs. 128/178	**OR: 0.33**	**0.12 to 0.86, 0%**
	**MCID at 6 months**	11/13 vs. 101/123	OR: 0.86	0.19 to 3.89, 0%
	**SEPQ3 at 1 month**	28 vs. 180	MD: −7.2	−15 to 0.87, 40%
	**SEPQ3 at 3 months**	28 vs. 178	MD: 1.5	−7.9 to 11, 0%
	**SEPQ3 at 6 months**	13 vs. 123	MD: −2.2	−21 to 17, 0%
	**RI at 3 months**	11 vs. 77	MD: 0	−0.04 to 0.04, 0%

*Note*: Bold cells indicate statistical significance.

Abbreviations: CAD, coronary artery disease; DM, diabetes mellitus; GRADE, grading of recommendations assessment, development and evaluation; IIEF‐EF, International Index of Erectile Function—Erectile Function domain; MCID, minimal clinically important difference; MD, mean difference; OR, odds ratio; RI, resistance index; SEPQ3, Question 3 of the sexual encounter profile diaries.

In the subgroup analysis based on the number of sessions, 6 versus 12 LiST sessions displayed similar efficacy in patients with DM or CAD versus other causes of vasculogenic ED. No adverse events were reported during the treatment protocol. The importance of these findings was considered critical, and the overall strength of evidence was deemed high for all outcomes based on the GRADE approach. Nevertheless, some outcomes may be underpowered, given that these analyses were performed with a relatively small sample size (e.g., *n* = 13 for CAD at 6 months). Another important limitation of the present study is that our findings may lack generalizability since we summarized the results of RCTs from a single center. Still, our decision to conduct an IPD meta‐analysis using RCTs from a single academic center was based on the unique opportunity to analyze participant‐level data from methodologically homogeneous studies that applied an identical LiST protocol.

It should be highlighted that LiST should be only considered for patients with vasculogenic ED. Even though DM and CAD are the commonest causes of vasculogenic ED, studies exploring the role of LiST on these patients are scarce.^13^ Our findings suggest that, irrespective of the underlying vascular damage, LiST may be effective. The latter may be attributed to the proposed mechanisms of LiST, including angiogenesis, activation of the endothelial progenitor cells, and, in turn, improved penile hemodynamics.^14^ Importantly, our findings are in line with previous meta‐analyses, reporting significant improvements in erectile function following LiST.^15^ Still, these studies could not specifically assess outcomes solely in patients with DM or CAD, highlighting the added value of our IPD approach.

The findings of this IPD meta‐analysis based on double‐blind RCTs deriving from one center of expertize that applied a standardized protocol indicate that LiST is equally safe and effective in patients with vasculogenic ED due to DM or CAD compared to other causes of ED. The insights from these high‐quality RCTs emphasize the beneficial role of LiST in patients with different degrees and causes of vasculogenic ED. In particular, 6 or 12 LiST sessions with the ARIES 2 LiST generation applying 5000 impulses with an energy level of 7 improve vasculogenic ED due to DM or CAD. In conclusion, LiST may be discussed with all patients with vasculogenic ED, irrespective of its initial cause.

## AUTHOR CONTRIBUTIONS

All authors participated in the drafting, writing, and editing of the manuscript.

## FUNDING INFORMATION

This research did not receive any specific grant from funding agencies in the public, commercial, or not‐for‐profit sectors.

## Data Availability

The data supporting this study's findings are available from the corresponding author upon reasonable request.

## References

[1] Sokolakis I , Hatzichristodoulou G . Clinical studies on low intensity extracorporeal shockwave therapy for erectile dysfunction: a systematic review and meta‐analysis of randomised controlled trials. Int J Impot Res. 2019;31:177‐194. doi:10.1038/s41443-019-0117-z 30664671

[2] Pyrgidis N , Kalyvianakis D , Mykoniatis I , Hatzichristou D . The recommended treatment protocol for low‐intensity shockwave therapy based on the severity of erectile dysfunction. Int J Impot Res. 2024. doi:10.1038/s41443-024-00959-7 Epub ahead of print. PMID: 39107452. PMC12283408 39107452

[3] Salonia A , Bettocchi C , Boeri L , et al. European Association of Urology Guidelines on Sexual and Reproductive Health‐2021 Update: male sexual dysfunction. Eur Urol. 2021;S0302‐2838(21):01813‐01813. doi:10.1016/j.eururo.2021.06.007 34183196

[4] Defeudis G , Mazzilli R , Tenuta M , et al. Erectile dysfunction and diabetes: a melting pot of circumstances and treatments. Diabetes Metab Res Rev. 2022;38:e3494. doi:10.1002/dmrr.3494 34514697 PMC9286480

[5] Farmakis IT , Pyrgidis N , Doundoulakis I , Mykoniatis I , Akrivos E , Giannakoulas G . Effects of major antihypertensive drug classes on erectile function: a network meta‐analysis. Cardiovasc Drugs Ther. 2022;36(5):903‐914. doi:10.1007/s10557-021-07197-9 Epub 2021 May 4. PMID: 33945044. 33945044

[6] Pyrgidis N , Mykoniatis I , Haidich A‐B , et al. The effect of phosphodiesterase‐type 5 inhibitors on erectile function: an overview of systematic reviews. Front Pharmacol. 2021;12:735708. doi:10.3389/fphar.2021.735708 34557099 PMC8452927

[7] Rosen RC , Allen KR , Ni X , Araujo AB . Minimal clinically important differences in the erectile function domain of the International Index of Erectile Function scale. Eur Urol. 2011;60:1010‐1016. doi:10.1016/j.eururo.2011.07.053 21855209

[8] Kalyvianakis D , Memmos E , Mykoniatis I , Kapoteli P , Memmos D , Hatzichristou D . Low‐intensity shockwave therapy for erectile dysfunction: a randomized clinical trial comparing 2 treatment protocols and the impact of repeating treatment. J Sex Med. 2018;15:334‐345. doi:10.1016/j.jsxm.2018.01.003 29396020

[9] Kalyvianakis D , Mykoniatis I , Memmos E , Kapoteli P , Memmos D , Hatzichristou D . Low‐intensity shockwave therapy (LiST) for erectile dysfunction: a randomized clinical trial assessing the impact of energy flux density (EFD) and frequency of sessions. Int J Impot Res. 2020;32:329‐337. doi:10.1038/s41443-019-0185-0 31474753

[10] Mykoniatis I , Pyrgidis N , Zilotis F , et al. The effect of combination treatment with low‐intensity shockwave therapy and tadalafil on mild and mild‐to‐moderate erectile dysfunction: a double‐blind, randomized, placebo‐controlled clinical trial. J Sex Med. 2022;19:106‐115. doi:10.1016/j.jsxm.2021.10.007 34866029

[11] Kalyvianakis D , Mykoniatis I , Pyrgidis N , et al. The effect of low‐intensity shock wave therapy on moderate erectile dysfunction: a double‐blind, randomized, sham‐controlled clinical trial. J Urol. 2022;208:388‐395. doi:10.1097/JU.0000000000002684 35830338

[12] Kalyvianakis D , Mykoniatis I , Pyrgidis N , Kapoteli P , Zilotis F , Hatzichristou D . The effect of combination treatment with low‐intensity shockwave therapy and daily tadalafil on severe erectile dysfunction: a double‐blind, randomized, sham‐controlled clinical trial. J Sex Med. 2024:qdae038. doi:10.1093/jsxmed/qdae038 38600694

[13] Mason MM , Pai RK , Masterson JM , Lokeshwar SD , Chu KY , Ramasamy R . Low‐intensity extracorporeal shockwave therapy for diabetic men with erectile dysfunction: a systematic scoping review. Andrology. 2023;11:270‐281. doi:10.1111/andr.13197 35642619

[14] Sokolakis I , Dimitriadis F , Teo P , Hatzichristodoulou G , Hatzichristou D , Giuliano F . The basic science behind low‐intensity extracorporeal shockwave therapy for erectile dysfunction: a systematic scoping review of pre‐clinical studies. J Sex Med. 2019;16:168‐194. doi:10.1016/j.jsxm.2018.12.016 30770067

[15] Lu Z , Lin G , Reed‐Maldonado A , Wang C , Lee Y‐C , Lue TF . Low‐intensity extracorporeal shock wave treatment improves erectile function: a systematic review and meta‐analysis. Eur Urol. 2017;71:223‐233. doi:10.1016/j.eururo.2016.05.050 27321373

